# Reversion from prediabetes to normoglycaemia and risk of cardiovascular disease and mortality: the Whitehall II cohort study

**DOI:** 10.1007/s00125-019-4895-0

**Published:** 2019-05-23

**Authors:** Dorte Vistisen, Mika Kivimäki, Leigh Perreault, Adam Hulman, Daniel R. Witte, Eric J. Brunner, Adam Tabák, Marit E. Jørgensen, Kristine Færch

**Affiliations:** 1 Department of Clinical Epidemiology, Steno Diabetes Center Copenhagen, Niels Steensens Vej 6, DK-2820 Gentofte, Denmark; 2 Department of Epidemiology and Public Health, University College London, 1–19 Torrington Place, London, WC1E 7HB UK; 3 Department of Medicine, Division of Endocrinology, Metabolism and Diabetes, University of Colorado, Anschutz Medical Campus, Aurora, CO USA; 4 Department of Public Health, Aarhus University, Aarhus, Denmark; 5 Danish Diabetes Academy, Odense, Denmark; 6 Steno Diabetes Center Aarhus, Aarhus, Denmark; 7 First Department of Medicine, Faculty of Medicine, Semmelweis University, Budapest, Hungary; 8 The Research Department for Health and Morbidity in the Population, Southern Denmark University, Copenhagen, Denmark

**Keywords:** 2 h Plasma glucose, Cardiovascular disease, Fasting plasma glucose, HbA_1c_, Mortality, Normoglycaemia, Prediabetes, Reversion

## Abstract

**Aims/hypothesis:**

Reversion from prediabetes to normoglycaemia is accompanied by an improvement in cardiovascular risk factors, but it is unclear whether this translates into a reduction in risk of cardiovascular disease (CVD) events or death. Hence, we studied the probability of reversion from prediabetes to normoglycaemia and the associated risk of future CVD and death using data from the Whitehall II observational cohort study.

**Methods:**

Three glycaemic criteria for prediabetes (fasting plasma glucose [FPG] 5.6–6.9 mmol/l, 2 h plasma glucose [2hPG] 7.8–11.0 mmol/l, and HbA_1c_ 39–47 mmol/mol [5.7–6.4%]) were assessed in 2002–2004 and 2007–2009 for 5193 participants free of known diabetes at enrolment. Among participants with prediabetes in the first examination, we calculated the probability of reversion to normoglycaemia by re-examination according to each glycaemic criterion. Poisson regression analysis was used to estimate and compare incidence rates of a composite endpoint of a CVD event or death in participants with prediabetes who did vs did not revert to normoglycaemia. Analyses were adjusted for age, sex, ethnicity and previous CVD.

**Results:**

Based on the FPG criterion, 820 participants had prediabetes and 365 (45%) of them had reverted to normoglycaemia in 5 years. The corresponding numbers were 324 and 120 (37%) for the 2hPG criterion and 1709 and 297 (17%) for the HbA_1c_ criterion. During a median follow-up of 6.7 (interquartile range 6.3–7.2) years, 668 events of non-fatal CVD or death occurred among the 5193 participants. Reverting from 2hPG-defined prediabetes to normoglycaemia vs remaining prediabetic or progressing to diabetes was associated with a halving in event rate (12.7 vs 29.1 per 1000 person-years, *p* = 0.020). No association with event rate was observed for reverting from FPG-defined (18.6 vs 18.2 per 1000 person-years, *p* = 0.910) or HbA_1c_-defined prediabetes to normoglycaemia (24.5 vs 22.9 per 1000 person-years, *p* = 0.962).

**Conclusions/interpretation:**

Most people with HbA_1c_-defined prediabetes remained prediabetic or progressed to diabetes during 5 years of follow-up. In contrast, reversion to normoglycaemia was frequent among people with FPG- or 2hPG-defined prediabetes. Only reversion from 2hPG-defined prediabetes to normoglycaemia was associated with a reduction in future risk of CVD and death.

**Electronic supplementary material:**

The online version of this article (10.1007/s00125-019-4895-0) contains peer-reviewed but unedited supplementary material, which is available to authorised users.

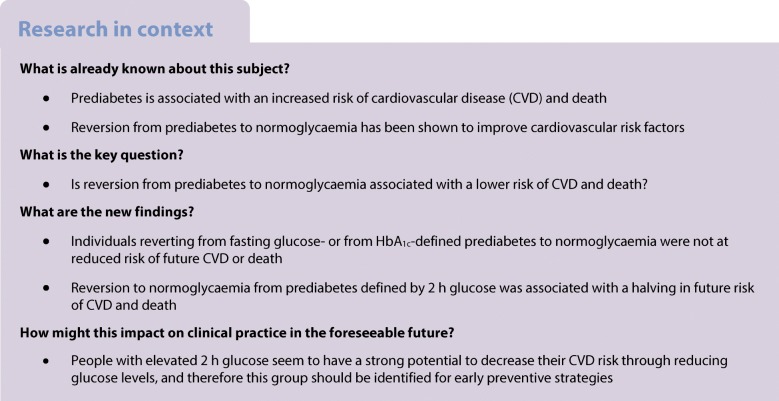



## Introduction

Prediabetes increases the risk of cardiovascular disease (CVD) and mortality [1, 2], and reversion from prediabetes to normoglycaemia is related to an improvement in a range of cardiovascular risk factors [3]. However, whether reversion translates into a reduction in risk of CVD or death has yet to be determined. To fill this knowledge gap, the current analysis explored the probability of reversion from prediabetes to normoglycaemia from different definitions of prediabetes and the associated risk of future CVD and death in a longitudinal follow-up of the well-described Whitehall II observational cohort study.

## Methods

### Study population

The Whitehall II study is an occupational cohort of 10,308 British civil servants who have been followed up with clinical examinations every 5 years since 1985 [4]. The current analysis utilised data from phase 7 (2002–2004) and phase 9 (2007–2009), when fasting plasma glucose (FPG), 2 h plasma glucose (2hPG) and HbA_1c_ were measured. From the 6967 participants at phase 7, we excluded 671 (9.6%) who did not participate at phase 9, 392 (5.6%) with known diabetes at phase 7, 684 (9.8%) who could not be classified with normoglycaemia, prediabetes or diabetes at both phases on at least one criterion, and 27 (0.4%) with screen-detected diabetes according to all the three criteria at phase 7, leaving 5193 (74.5%) of the phase 7 participants for analysis (ESM Fig. 1). For the analyses of FPG and 2hPG, participants had to have fasted for ≥8 h before the clinical examinations. The University College London Ethics Committee reviewed and approved the study. Written informed consent was obtained from all participants at each study phase.

### Measurements and definitions

Participants underwent a standard 75 g OGTT with measurement of FPG and 2hPG using the glucose oxidase method [4]. HbA_1c_ was measured in whole blood, drawn into BD Vacutainers (Becton Dickinson, Winnersh, UK), using the validated Tosoh G8 high-performance ion-exchange liquid chromatography platform (Tosoh Bioscience, Tessenderlo, Belgium).

Prediabetes was defined as FPG 5.6–6.9 mmol/l, 2hPG 7.8–11.0 mmol/l and/or HbA_1c_ 39–47 mmol/mol (5.7–6.4%) according to criteria from the ADA [5]. For each of the three criteria, normoglycaemia and diabetes were defined as values below and above the cut-off points for prediabetes, respectively. Diabetes could also be diagnosed by a doctor outside the study.

The primary outcome was a composite endpoint of a CVD event or death between 2007–2009 (phase 9) and the end of follow-up (30 June 2015). Adjudicated CVD events included fatal and non-fatal myocardial infarction and stroke [2]. All-cause mortality was obtained from the NHS Central Registry, which provided information on the cause and date of death.

### Statistical analysis

All analyses were conducted separately for the three glycaemic criteria, in accordance with the guidelines from ADA [5] and the International Expert Committee (IEC) [6], which suggest that the different criteria to define prediabetes and diabetes should not be combined. For each criterion, participants with screen-detected diabetes at baseline by the given glycaemic criterion were excluded.

Among individuals with prediabetes at phase 7, we calculated the probability of reversion to normoglycaemia at the phase 9 re-examination approximately 5 years later. Poisson regression analysis with log(person time) as offset was used to estimate and compare incidence rates of future CVD or death in individuals with prediabetes who did or did not revert to normoglycaemia. The follow-up period of each participant was split into 1 year age bands to account for the non-constant effect of age over time on CVD risk and mortality. Analyses were adjusted for age, sex, ethnicity and previous CVD. A complete case approach was used.

All participants with 2hPG available also had measurements of FPG and HbA_1c_ (but not vice versa). Among participants with 2hPG-defined prediabetes and elevated FPG and/or HbA_1c_, we compared event rates between participants who did or did not revert to normoglycaemia on FPG and/or HbA_1c_, without normalising 2hPG (ESM Fig.1).

We further estimated the associations of changes in FPG, 2hPG or HbA_1c_ from phase 7 to phase 9 with risk of future CVD or death, excluding participants with known diabetes at phase 9 because they were likely to be receiving treatment.

Statistical analyses were performed in R, version 3.4.1 (R Foundation for Statistical Computing, Vienna, Austria).

## Results

The study population was predominantly men (73%) and of white ethnicity (93%), 769 (15%) had pre-existing CVD, and the average age was 60 years (range 50–73 years) at the first clinical examination (phase 7). During a median follow-up of 6.7 (interquartile range 6.3–7.2) years, 668 events of non-fatal CVD or death occurred among the 5193 participants. Comparing normoglycaemia vs prediabetes by each glycaemic criterion (Table 1), more men than women had prediabetes defined by FPG whereas no sex difference was observed for prediabetes defined by 2hPG or HbA_1c_. Participants with 2hPG- or HbA_1c_-, but not FPG-defined prediabetes were older (*p* < 0.001) and more likely to be of non-white ethnicity (*p* ≤ 0.044) compared with their normoglycaemic counterparts.

**Table 1:** Characteristics of study participants at the first clinical examination (phase 7) by glycaemic criterion

Characteristic	FPG criterion		2hPG criterion		HbA_1c_ criterion	
	Normoglycaemia	Prediabetes	Normoglycaemia	Prediabetes	Normoglycaemia	Prediabetes
*n*	2130	820	2154	324	3337	1709
Men (%)	70.0 (68.1, 72.0)	83.9 (81.2, 86.4)	75.7 (73.8, 77.5)	76.2 (71.2, 80.8)	72.6 (71.0, 74.1)	73.6 (71.5, 75.7)
White ethnicity (%)	92.5 (91.3, 93.6)	93.2 (91.2, 94.8)	93.1 (91.9, 94.1)	89.8 (86.0, 92.9)	96.3 (95.6, 96.9)	89.4 (87.9, 90.8)
Age (years)	60.2 ± 5.8	60.6 ± 5.8	59.6 ± 5.6	62.0 ± 6.1	60.2 ± 5.7	61.6 ± 5.9
BMI (kg/m^2^)	24.0 ± 3.1	24.9 ± 3.2	24.0 ± 3.1	24.9 ± 3.1	23.9 ± 3.0	24.6 ± 3.3
Total cholesterol (mmol/l)	5.7 ± 1.0	5.8 ± 1.0	5.7 ± 1.0	5.7 ± 1.0	5.7 ± 1.0	5.8 ± 1.0
HDL-cholesterol (mmol/l)	1.6 ± 0.5	1.5 ± 0.4	1.6 ± 0.4	1.5 ± 0.4	1.6 ± 0.5	1.5 ± 0.4
LDL-cholesterol (mmol/l)	3.5 ± 0.9	3.6 ± 0.9	3.6 ± 0.9	3.6 ± 0.9	3.5 ± 0.9	3.6 ± 0.9
Triacylglycerols (mmol/l)	1.1 (0.8–1.5)	1.2 (0.9–1.7)	1.1 (0.8–1.5)	1.3 (0.9–1.85)	1.1 (0.8–1.5)	1.2 (0.9–1.8)
Systolic BP (mmHg)	126.0 ± 16.3	131.2 ± 16.4	125.6 ± 15.7	130.6 ± 16.7	126.3 ± 16.1	129.0 ± 16.3
Diastolic BP (mmHg)	73.6 ± 10.3	76.5 ± 10.1	73.7 ± 10.3	75.0 ± 10.2	73.8 ± 10.4	74.9 ± 10.2
FPG (mmol/l)	5.0 ± 0.3	5.9 ± 0.3	5.3 ± 0.5	5.6 ± 0.7	5.2 ± 0.5	5.5 ± 0.6
2hPG (mmol/l)	5.9 ± 1.5	6.9 ± 2.0	5.7 ± 1.1	8.9 ± 0.9	5.9 ± 1.5	6.6 ± 1.9
HbA_1c_ (mmol/mol)	33.0 ± 4.0	35.0 ± 5	33.0 ± 4	36.0 ± 5	35.0 ± 3.0	42.0 ± 2.0
HbA_1c_ (%)	5.2 ± 0.4	5.4 ± 0.4	5.2 ± 0.4	5.4 ± 0.4	5.4 ± 0.3	6.0 ± 0.2
Previous CVD (%)	12.4 (11.1, 13.9)	17.1 (14.6, 19.8)	12.5 (11.2, 14)	18.5 (14.4, 23.2)	11.4 (10.4, 12.6)	18.0 (16.2, 19.9)
Family history of DM (%)	9.3 (8.1, 10.6)	12.3 (10.1, 14.8)	9.1 (7.9, 10.4)	10.0 (6.9, 13.8)	8.4 (7.4, 9.4)	12.2 (10.7, 13.9)
Current smoker (%)	8.0 (6.9, 9.2)	7.9 (6.2, 10.0)	7.7 (6.6, 8.9)	4.6 (2.6, 7.5)	6.0 (5.2, 6.9)	9.2 (7.9, 10.7)
Alcohol intake (units/week)	8.0 (2.0–16.0)	12.0 (5.0–21.0)	10.0 (4.0–18.0)	9.0 (3.0–17.0)	10.0 (4.0–18.0)	8.0 (2.0–16.0)
Antihypertensive treatment (%)	19.5 (17.9, 21.3)	28.2 (25.1, 31.4)	18.4 (16.8, 20.1)	33.3 (28.2, 38.8)	17.9 (16.6, 19.3)	26.6 (24.5, 28.8)
Lipid-lowering treatment (%)	7.8 (6.7, 9.0)	13.4 (11.2, 15.9)	7.5 (6.4, 8.7)	15.4 (11.7, 19.8)	6.9 (6.0, 7.8)	13.4 (11.8, 15.1)

Data are means±SD, medians (25–75% percentiles; IQR) or proportions (95% CI)

HbA_1c_: normoglycaemia: <39 mmol/mol (5.7%), prediabetes 39–47 mmol/mol (5.7–6.4%), Fasting plasma glucose: normoglycaemia: <5.6 mmol/l, prediabetes 5.6–6.9 mmol/l, 2hPG: normoglycaemia: <7.8 mmol/l, prediabetes 7.8–11.0 mmol/l

DM, diabetes mellitus

### FPG criterion

**Fig. 1:**
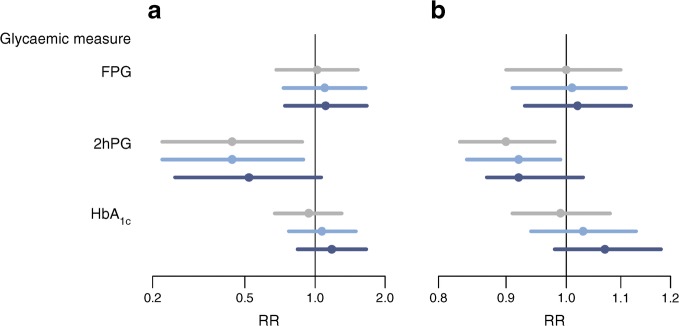
Rate ratios (RRs) of an event (CVD or death) for reverting from prediabetes to normoglycaemia vs not reverting (**a**) or for decreasing 1 SD in glycaemic measure over 5 years from phase 7 to phase 9 (**b**). Grey: unadjusted RR; light blue: adjusting for age and sex; dark blue: further adjusting for previous CVD. The RR for 1 SD decrease (**b**) is further adjusted for baseline glycaemia in all the analyses. The *x*-axis is on a natural logarithmic scale

Among 820 participants with FPG-defined prediabetes at baseline, 365 (45%) reverted to normoglycaemia and 111 (14%) progressed to diabetes at the 5 year re-examination. From the 5 year re-examination, median (interquartile range) follow-up time was 6.7 (6.3–7.2) years, during which 96 (12%) developed CVD or died. Reverting from prediabetes to normoglycaemia was not associated with a difference in rate of developing CVD or dying vs remaining prediabetic or progressing to diabetes (18.6 vs 18.2 per 1000 person-years, *p* = 0.962) (Fig. 1a, ESM Table 1).

### 2hPG criterion

Among 324 participants with 2hPG-defined prediabetes at baseline, 120 (37%) reverted to normoglycaemia and 73 (23%) progressed to diabetes after 5 years. Follow-up time for CVD and death was 6.7 (6.4–7.2) years, during which 47 (15%) developed CVD or died. Reverting from prediabetes to normoglycaemia was associated with a halving of the event rate vs remaining prediabetic/progressing to diabetes (12.7 vs 29.1 per 1000 person-years, *p* = 0.020), and was largely unchanged after adjustment for age, sex, ethnicity and previous CVD (Fig. 1a, ESM Table 1).

Among the 324 participants with prediabetes at baseline, 220 also had prediabetes according to the FPG and/or HbA_1c_ criteria. At the 5 year re-examination, 21 (10%) of these had reverted to normoglycaemia according to the FPG and/or HbA_1c_ criteria but not according to the 2hPG criterion. However, improvement in FPG or HbA_1c_ without improvement in 2hPG was not associated with a lower event rate (32.1 vs 28.8 per 1000 person-years, respectively, *p* = 0.835).

### HbA_1c_ criterion

Among 1709 participants with HbA_1c_-defined prediabetes at baseline, 297 (17%) reverted to normoglycaemia and 233 (14%) progressed to diabetes after 5 years. Follow-up time for an event was 6.7 (6.3–7.2) years, during which 258 (15%) developed CVD or died. Reverting from prediabetes to normoglycaemia was not associated with a difference in rate of developing CVD or dying vs remaining prediabetic/progressing to diabetes (24.5 vs 22.9 per 1000 person-years, *p* = 0.962) (Fig. 1a, ESM Table 1).

The analysis of absolute changes in FPG, 2hPG or HbA_1c_ from phase 7 to phase 9 confirmed the results (Fig. 1b, ESM Table 2).

## Discussion

In this cohort study, reversion from 2hPG-defined prediabetes to normoglycaemia was associated with an approximately 50% lower risk of a CVD event or death compared with remaining with prediabetes or progressing to diabetes. Despite prediabetes being a known risk factor for CVD, we found that reversion from FPG- or HbA_1c_-defined prediabetes to normoglycaemia was not associated with a lower risk of CVD or death. Outside pregnancy, the OGTT has largely been eliminated for diagnosing diabetes, particularly with the recent widespread standardisation of the HbA_1c_ assay. However, our findings suggest that identification of people with elevated 2hPG should be considered for CVD risk stratification either by re-introducing the OGTT for diagnosing prediabetes and diabetes or by other methods [7, 8].

Our results support previous findings from the Diabetes Prevention Program (DPP), where individuals with 2hPG-defined prediabetes who reverted to normoglycaemia experienced a concomitant reduction in their cardiovascular risk profile [9]. Previous observations from a general Dutch population also suggested that 2hPG levels are more strongly associated with all-cause and cardiovascular mortality than FPG or HbA_1c_ levels in the non-diabetic range [10]. These results may reflect underlying pathophysiological differences in, for example, insulin resistance, which is more pronounced in elevated 2hPG than in elevated FPG and HbA_1c_ [11].

We found reversion to normoglycaemia from HbA_1c_-defined prediabetes to be less likely than from FPG- or 2hPG-defined prediabetes. While our results on reversion rates for FPG- and 2hPG-defined prediabetes are consistent with previous findings [12], evidence on the ability to revert from HbA_1c_-defined prediabetes to normoglycaemia is scarce [12]. A Japanese study reported a reversion rate of 32% in a study population that was, on average, 10 years younger than the participants of the Whitehall II study [12]. There is less day-to-day variation in HbA_1c_, and levels in the non-diabetic range may largely be explained by non-glycaemic factors, such as age and ethnicity [13], and may therefore not be modifiable to the same degree as FPG and 2hPG levels.

We have chosen not to combine different definitions of prediabetes and assess the impact of overall reverting to normoglycaemia. Combining definitions is not in accordance with ADA and IEC and will greatly inflate the prevalence of prediabetes [14]. Furthermore, the corresponding state of normoglycaemia needs to be defined as normal values on all three criteria, which may not be relevant in clinical practice. Alternatively, specific states of prediabetes and normoglycaemia will have to be applied, resulting in numerous transition possibilities for which this study is not powered to examine (ESM Fig. 2 and 3).

Results from the current analysis are strengthened by the longitudinal, well-described, large population size and the validated ascertainment of CVD events. Nevertheless, reversion cannot be ascribed to intervention effects, since the cohort is strictly observational, and all conclusions remain associative and not necessarily causative. There is still controversy between different diabetes organisations with respect to whether HbA_1c_ or FPG should be used to define prediabetes. Our results did not show a reduced risk of CVD or death when people with either HbA_1c_- or FPG-defined prediabetes were able to revert to normoglycemia, based on the respective criterion. On the other hand, we found reversion from 2hPG-defined prediabetes to be associated with a halving of the risk of CVD and death. Although the different diagnostic criteria for prediabetes are likely to remain, the current results would contend that only reversion from 2hPG-defined prediabetes to normoglycaemia is sensitive enough to detect cardiovascular benefit.

Guidelines for people with diabetes are increasingly comprehensive [5], and accordingly, the incidence of complications has dramatically decreased over the past 20 years [15]. In contrast, the lack of accepted guidelines for people with prediabetes has made the prevalence of diabetes-related complications now virtually identical for people with prediabetes vs those with diabetes [16]. The current findings highlight the reduced risk for CVD and death associated with reversion from 2hPG-defined prediabetes, specifically, to normoglycaemia. These findings have important implications for additional cardiovascular risk stratification and intervention in a landscape that has become increasingly controversial [17].

## Electronic supplementary material

ESM (PDF 287 kb)

## Data Availability

Whitehall II data, protocols and other metadata are available to the scientific community. Please refer to the Whitehall II data sharing policy at https://www.ucl.ac.uk/whitehallII/data-sharing.

## Abbreviations

2hPG: 2 h plasma glucose
CVD: Cardiovascular disease
FPG: Fasting plasma glucose
IEC: International Expert Committee
