# Supplementary material for: Reversion from prediabetes to normoglycaemia and risk of cardiovascular disease and mortality: the Whitehall II cohort study
Source: Diabetologia. 2019 May 23;62(8):1385–90. doi: 10.1007/s00125-019-4895-0 (PMC6647230; doi:10.1007/s00125-019-4895-0)
Supplement: Supplementary file 1 — (PDF 287 kb) [file 125_2019_4895_MOESM1_ESM.pdf]

Electronic supplemental material (ESM)

ESM Table 1 Rates and rate ratios of an event (cardiovascular disease or mortality) for prediabetes participants at phase 7 by glycaemic status at the phase 9 re-examination

**Prediabetes at first clinical examination (phase 7)**	**Glycaemic state at re-examination (phase 9)**	**N**	**No. events**	**Rate per 1000-PY**	**RR**	**RR_{adj1}**	**RR_{adj2}**
By FPG	Prediabetes or diabetes	455	53	18.2 (13.9, 23.8)	1.00 ref	1.00 ref	1.00 ref
	Normoglycaemia	365	43	18.6 (13.8, 25.1)	1.02 (0.68, 1.53)	1.10 (0.73, 1.65)	1.11 (0.74, 1.67)
By 2hPG	Prediabetes or diabetes	204	37	29.1 (21.1, 40.2)	1.00 ref	1.00 ref	1.00 ref
	Normoglycaemia	120	10	12.7 (6.82, 23.6)	0.44 (0.22, 0.88)	0.44 (0.22, 0.89)	0.52 (0.25, 1.06)
By HbA_{1c}	Prediabetes or diabetes	1412	216	22.9 (16.9, 31.0)	1.00 ref	1.00 ref	1.00 ref
	Normoglycaemia	297	42	24.5 (21.4, 28.0)	0.94 (0.67, 1.30)	1.07 (0.77, 1.50)	1.18 (0.84, 1.66)

FPG: fasting plasma glucose; 2hPG: 2-hour plasma glucose;

RR: crude rate ratio;

RR_{adj1}: rate ratio adjusted for age, sex and ethnicity;

RR_{adj2}: rate ratio further adjusted for previous CVD

ESM Table 2 Rates and rate ratios of an event (cardiovascular disease or mortality) for a unit decrease in glycaemic measure from phase 7 to phase 9 among participants with prediabetes or normoglycaemia at phase 7

**Glycaemic measure**		**N**	**No. events**	**RR**	**RR_{adj1}**	**RR_{adj2}**
FPG	(per 1 mmol/L)	2,819	326	1.00 (0.82, 1.21)	1.01 (0.83, 1.24)	1.04 (0.87, 1.25)
	(per 1 SD = 0.5 mmol/L)			1.00 (0.90, 1.10)	1.01 (0.91, 1.11)	1.02 (0.93, 1.12)
2hPG	(per 1 mmol/L)	2,396	258	0.93 (0.88, 0.99)	0.94 (0.89, 1.00)	0.96 (0.91, 1.02)
	(per 1 SD = 1.4 mmol/L)			0.90 (0.83, 0.98)	0.92 (0.84, 1.00)	0.95 (0.87, 1.03)
HbA_{1c}	(per 1 mmol/mol)	4,865	604	1.00 (0.98, 1.02)	1.00 (0.98, 1.03)	1.02 (0.99, 1.04)
	(per 1 SD = 3.9 mmol/mol)			0.99 (0.91, 1.08)	1.03 (0.94, 1.13)	1.07 (0.98, 1.18)

FPG: fasting plasma glucose; 2hPG: 2-hour plasma glucose;

RR: rate ratio adjusted for phase 7 glycaemic level;

RR_{adj1}: rate ratio further adjusted for age, sex and ethnicity;

RR_{adj2}: rate ratio further adjusted for previous CVD

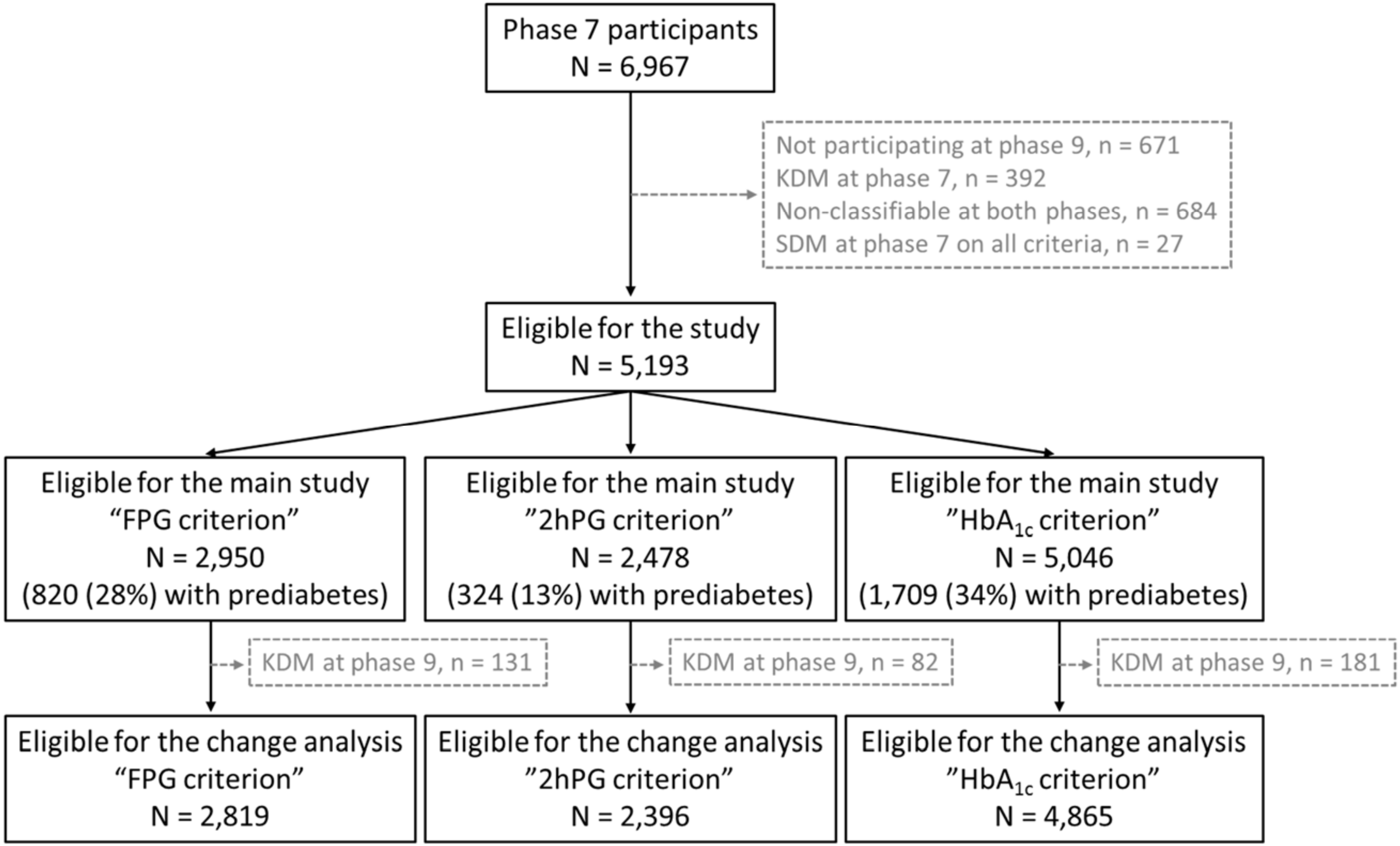

ESM Fig. 1 Flow chart of the study participants. KDM: known diabetes. SDM: screen detected diabetes. FPG: fasting plasma glucose. 2hPG: 2-hour plasma glucose

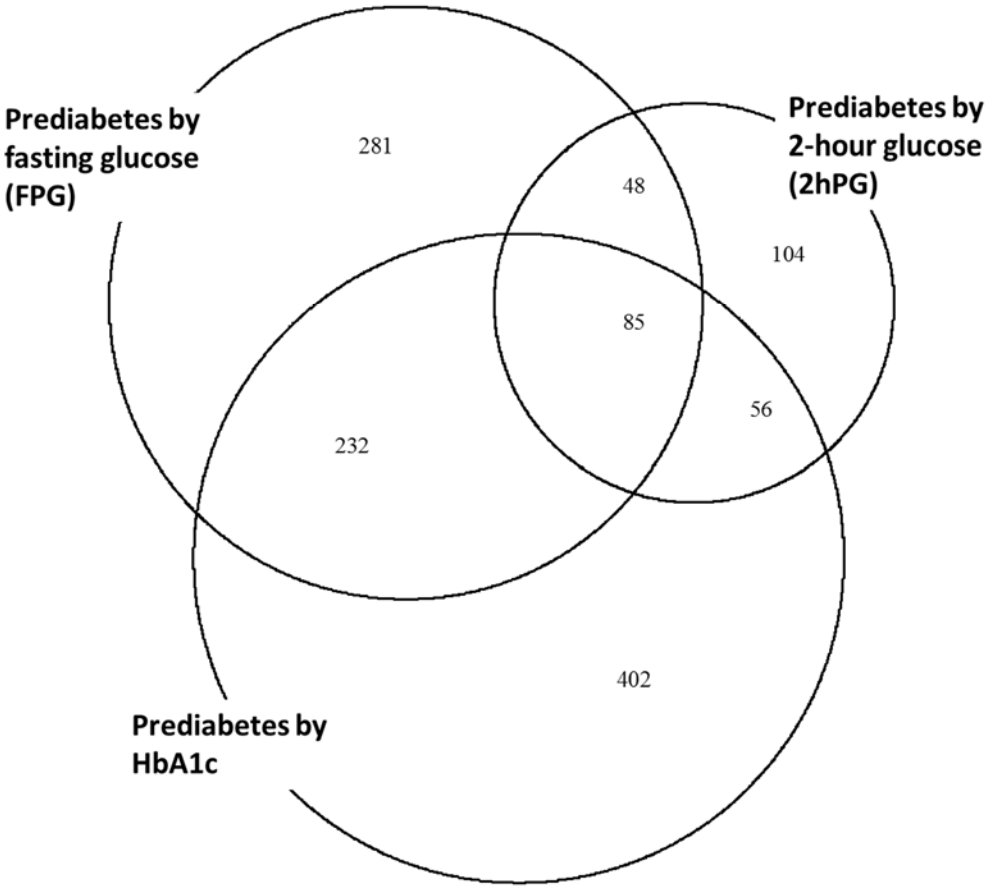

ESM Fig. 2 Venn diagram of the overlap between definitions of prediabetes. In the study population of 5,193 participants, 2,540 could be classified according to all three criteria (and did not have screen detected diabetes by any criteria). In total, 1,208 out of the 2,540 had prediabetes on at least one criterion, giving rise to seven different states of prediabetes

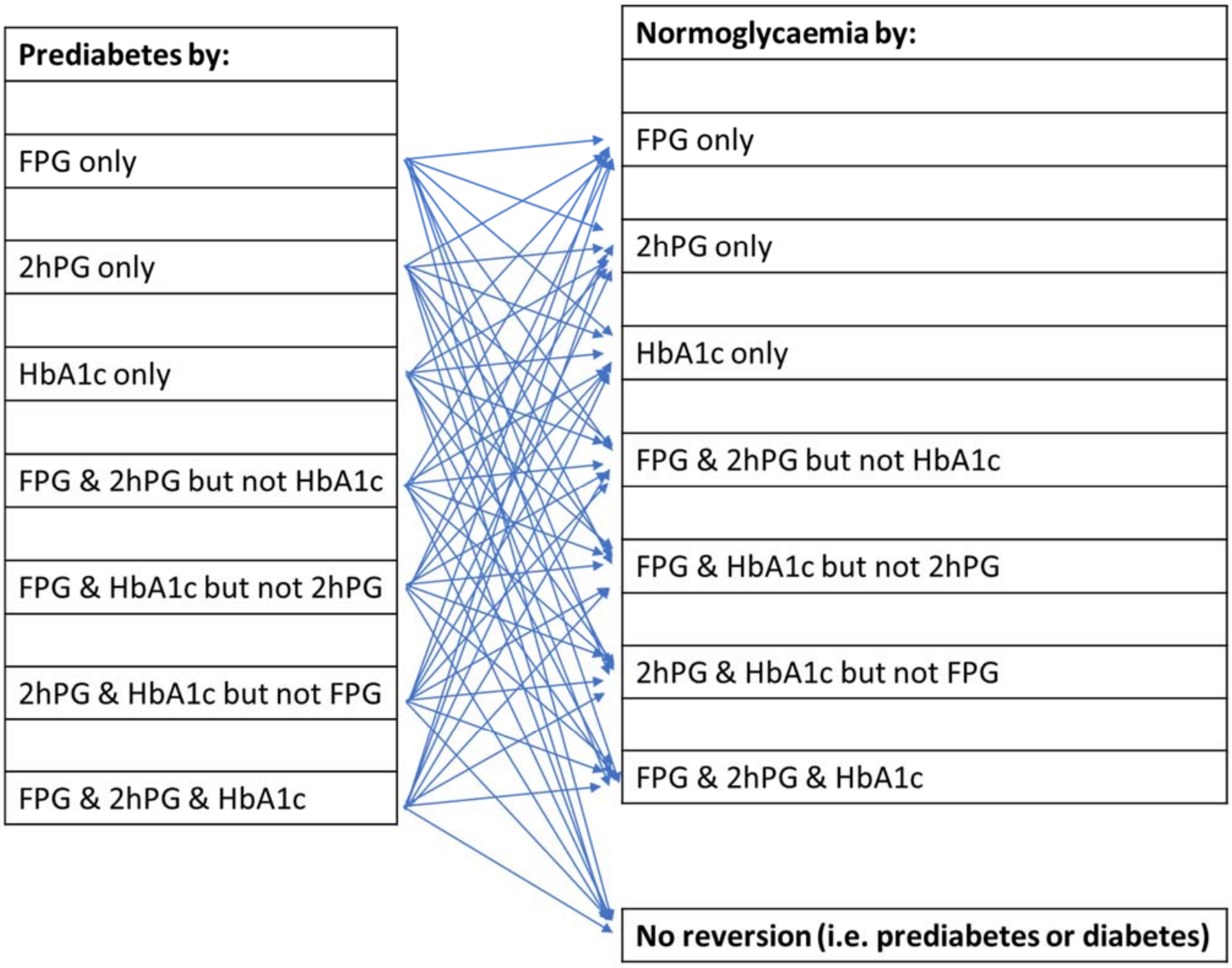

ESM Fig. 3 Transition possibilities from prediabetes to normoglycaemia or to no reversion. Participants in each of the seven prediabetes states can revert to one of seven different normoglycemic states or alternatively remain with prediabetes or progress to diabetes, giving rise to to a complex pattern of 56 transitions. FPG: fasting plasma glucose. 2hPG: 2 h plasma glucose.
